# Experimental determination of the weighting factor for the energy window subtraction–based downscatter correction for I-123 in brain SPECT studies

**DOI:** 10.4103/0971-6203.71765

**Published:** 2010

**Authors:** Robin de Nijs, Søren Holm, Gerda Thomsen, Morten Ziebell, Claus Svarer

**Affiliations:** Department of Clinical Physiology, Nuclear Medicine, PET and Cyclotron Unit, Copenhagen University Hospital, Copenhagen, Denmark; 1 Neurobiology Research Unit, Department of Neurology, Copenhagen University Hospital, Copenhagen, Denmark

**Keywords:** weighting factors, downscatter correction, SPECT, iodine-123, energy window subtraction

## Abstract

Correction for downscatter in I-123 SPECT can be performed by the subtraction of a secondary energy window from the main window, as in the triple-energy window method. This is potentially noise sensitive. For studies with limited amount of counts (e.g. dynamic studies), a broad subtraction window with identical width is preferred. This secondary window needs to be weighted with a factor higher than one, due to a broad backscatter peak from high-energy photons appearing at 172 keV. Spatial dependency and the numerical value of this weighting factor and the image contrast improvement of this correction were investigated in this study. Energy windows with a width of 32 keV were centered at 159 keV and 200 keV. The weighting factor was measured both with an I-123 point source and in a dopamine transporter brain SPECT study in 10 human subjects (5 healthy subjects and 5 patients) by minimizing the background outside the head. Weighting factors ranged from 1.11 to 1.13 for the point source and from 1.16 to 1.18 for human subjects. Point source measurements revealed no position dependence. After correction, the measured specific binding ratio (image contrast) increased significantly for healthy subjects, typically by more than 20%, while the background counts outside of all subjects were effectively removed. A weighting factor of 1.1–1.2 can be applied in clinical practice. This correction effectively removes downscatter and significantly improves image contrast inside the brain.

## Introduction

**Figure 1:**
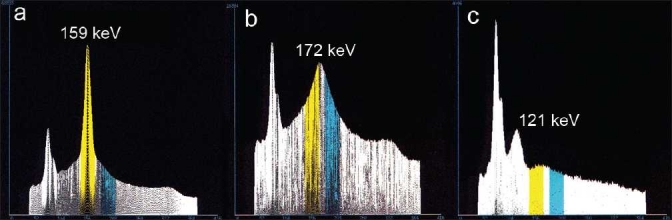
Energy spectra of an iodine-123 point source measured by the SPECT-scanner: (a) unshielded I-123 source in air; (b) source shielded with 6-mm lead, showing a broad peak at 172 keV due to reflected high-energy photons; (c) source placed outside the scanner, showing a peak at 121 keV from 90° Compton scattering of 159 keV photons inside the scanner. The main energy window is highlighted with yellow and the second energy window with cyan

Radioisotopes used for imaging with single-photon emission computer tomography (SPECT), such as Technetium-99m, typically emit photons with energy between 100 and 200 keV. This allows effective collimation by lead. These emissions have a photon energy of 159 keV for Iodine-123, which also emits a significant amount of photons with a higher energy (abundance 3.1%). The most important of these have a photon energy of 529 keV and an abundance of 1.4%, whereas the primary photons at 159 keV have an abundance of 83.4%.[1] The high-energy photons are not well collimated, but penetrate through the lead into the scintillation crystal (septal penetration). Photons from parts of the patient’s body outside the SPECT scanner may contribute to the projection data by penetration through either the collimator or the camera shield. Compton scatter for 529 keV photons in the sodium iodide crystal of the detector is a far more likely process than photo absorption, and only a minor portion of the counts will be located at the 529 keV photopeak. The resulting photons from scatter in the object will be collimated and are therefore less important than the high-energy photons scattering in the detector. Because the high-energy photons are detected at a lower energy, this process is called downscatter. The resulting spectrum is rather flat above the 159 keV peak, except for a weak and broad backscatter peak at 172 keV. This peak corresponds to photons passing the collimator and crystal without interaction, while being reflected in the material behind the crystal and finally being absorbed. Due to the low efficiency of collimators and the high probability of penetration, the erroneous counts coming from the high-energy photons become a significant fraction of the observed signal at 159 keV, despite their lower abundance. Consequently, the reconstructed SPECT images are deteriorated. The contribution of the high-energy photons depends on the type of collimator and can be significant for low-energy collimators,[2] as demonstrated by the fact that the so-called Compton edge at 358 keV (coming from the 529 keV photons) is visible in the energy spectrum [Figure 1a].

The observation of a fairly constant energy spectrum above the 159 keV peak [Figure 1a] suggests that a correction can be estimated from a second energy window with an identical width above the one used for imaging. If a flat energy spectrum is assumed, then the second energy window is just subtracted from the main window before reconstruction.[3–5] However, within the main window at 172 keV, a broad backscatter peak, coming from high-energy photons, is present.[6] Therefore, it is expected that the second energy window has to be weighted with a factor slightly higher than one, depending on the height of the backscatter peak.

The triple-energy window (TEW) method[7] also corrects for downscatter but is based on the subtraction of narrow abutting energy windows, which are potentially noise sensitive in SPECT studies with limited counts (e.g., dynamic). The photons in the narrow abutting window, however, mimic the downscattered photons better than a broad energy window. This is also true for the correction of scatter of the main photons. The scatter correction part of the TEW method mimics scattered main photons better than the broad scatter correction window in the dual-energy window method[8], while the latter is potentially less noise sensitive. Other mathematically more advanced scatter correction methods[9,23] exist, but they demand more sophisticated ways of postprocessing and reconstruction. A downscatter correction by subtracting a uniform off-set[10,11] does not take the difference between spatial distribution of the downscattered and primary photons into account.

The impact of the energy window subtraction on the final images, and the value of the appropriate weighting factor for the second energy window, have been investigated in a brain SPECT study and in experiments with an I-123 point source. The kinetics are influenced by scatter corrections,[12] but this issue is not addressed here.

## Materials and Methods

### Energy windows and imaging

Measurements were performed with a triple-head IRIX camera (Philips Medical, Cleveland, U.S.A.) fitted with parallel hole, low-energy, general purpose (LEGP) collimators (spatial resolution 8.5 mm at 10 cm distance to the collimator) with an orbit radius of 16.5 cm. Projection data were obtained in 128 × 128 matrix size with an isotropic pixel size of 2.33 mm.

The energy window for the primary imaging photons, with corresponding raw projection data I, was set at 143–175 keV. The energy window for the downscattered photons with projection data D was set at 184–216 keV. Both energy windows have a full width of 32 keV. In order to minimize the contamination of the downscatter window by primary photons (caused by limited energy resolution) a small energy gap (175–184 keV) between the two windows was chosen. Downscatter-corrected projection data J was calculated using the formula:

(1) J=I−k·D

where *k* is the spatially invariant weighting factor for the downscatter window.[3–5]

**Figure 2:**
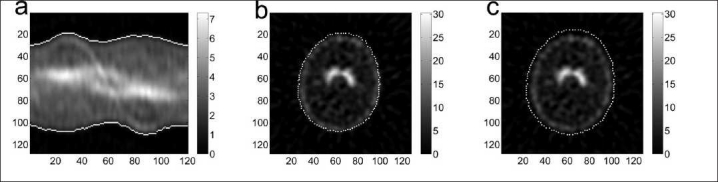
Illustration of the edge detection in a healthy subject (subject 3). The left panel (a) shows the so-called sinogram for a slice. Vertically the tangential position, and horizontally the 120 angles, are shown. The middle panel (b) shows the corresponding reconstructed slice. The edge is shown with maximum intensity (white). The right panel (c) shows the edge with a radial offset of 3 pixels.

SPECT imaging was performed by recording projection data at 120 fixed angles, with an interval of 3° and a noncircular orbit. The mean radius of rotation was 13.9 cm. Reconstruction of projection data with standard filtered back-projection (FBP), both with and without downscatter correction, was performed in MATLAB 7.5 (Mathworks, USA). Matrix size was 128 × 128, with 2.33 mm pixels and identical slice thickness. A 3D low-pass 4^th^-order Butterworth post-filter with a cut-off frequency of 0.3 Nyquist (= 0.64 cm^–1^) was used. Attenuation correction with Chang’s first-order correction[13] was applied with an empirical linear attenuation factor of 0.10 cm^–1^ for I-123 imaging without Compton scatter correction of the primary 159 keV photons.[11,14–16] The determination of the attenuation map was aided by an algorithm, which finds the most outward placed crossing of a manually set threshold (tuned at the edge) and the intensity for every projection angle in the sinogram [Figure 2a and b].

### Experimental determination of the weighting factor

The weighting factor *k* in Equation 1 was determined experimentally with an I-123 point source with 37 MBq activity. The contribution of the primary photons was ‘removed’ either: (i) by shielding the source with 6-mm lead, thus efficiently excluding the 159 keV photons, and placing the source inside the camera field-of-view (FOV) or (ii) by placing the unshielded I-123 source outside the FOV, near the scanner axis but 20 cm in front of the gantry and placing a 6.3-liter cylindrical water-filled phantom with a diameter of 16 cm inside the scanner as a scattering medium for mimicking a subject.

The camera heads in both experiments were positioned at 90° (collimator surface perpendicular to the floor), 210°, and 330°, and the weighting factor *k* was determined as the ratio of the total counts between the two energy windows in the raw projection data, using the formula:

(2) $$k\;=\;\frac{\sum I}{\sum D}$$

for each angle. The energy spectrum in Figure 1b shows the contribution of the high-energy photons for the lead-shielded I-123 point source (method I) as a broad peak at 172 keV. This energy spectrum is similar to the energy spectrum of a Fluor-18 source (emitting mono-energetic photons of 511 keV) in the SPECT scanner with low-energy collimators. In method II, some of the 159 keV primary photons scatter around 90° in the water-filled phantom and give rise to 121 keV photons, while the primary photons are effectively removed [Figure 1c]. The broad backscatter peak of the high-energy photons at 172 keV was less visible in the latter case.

In order to investigate the spatial dependence of the weighting factor *k*, pixelwise *k*-maps were calculated. The statistical uncertainty in *k* depends on the number of counts. Therefore, a *z*-score, which does not scale with the amount of counts, was calculated. With *I* and *D* the pixel value in the corresponding projection data *I* and *D* the *z*-score for each pixel was defined by the difference between the global *k* factor defined by Equation 2 and the pixel value *k*_pixel_= *I/D*, normalized by the theoretical standard deviation σ_pixel_; this was given by the formula

(3) $$z\;=\;\frac{k\;-\;k_{\mathrm{pixel}}}{\sigma_{\mathrm{pixel}}}\approx \frac{k\;-\;\frac{1}{D}}{k\cdot \sqrt{\frac{1}{I}+\frac{1}{D}}}$$

On the right hand side, the variance (σ^2^) of the ratio *I/D* was approximated, for small variances, by adding the relative variances of *I* and *D*. These were expressed, assuming Poisson statistics, as the reciprocal value of the number of counts.

In order to detect significant outliers, the double-sided *P*-value was calculated from z for every pixel, based on the assumption that the data was normally distributed. Negative differences in Equation 3 were indicated with a negative *P*-value. All pixelwise *P*-values were corrected for multiple comparisons with the Bonferroni method, by multiplication with a global scaling factor $P/\left(1-\sqrt[{n}]{1-P}\right),$, where *P*=.05 is the overall significance level and n the number of pixels. For small *P*-values, the scaling factor is approximately equal to the number of comparisons. Thus, a scaled *P*-value of .05 corresponds to a double-sided 5% confidence value corrected for multiple comparisons. Since outliers have a low *P*-value, pixelwise 1/*P* maps were calculated. Pixelwise *k*-maps and 1/*P*-maps were investigated in different matrix sizes because a different balance between uncertainty and spatial resolution can reveal other outliers. Resolution was reduced by box filtering with a uniform kernel, i.e., adding the counts in the kernel area. In this way, Poisson statistics were preserved at the reduced resolution.

### Subjects and evaluation of the downscatter correction

The downscatter correction was investigated in human brain studies of the dopamine transporter binding. Five subjects with a high specific binding ratio are referred to as healthy subjects, while five subjects with low specific binding ratio are referred to as patients. All subjects gave their informed written consent and the study was performed in accordance with the ethical standards set out in the Declaration of Helsinki and was approved by the ethical committee of Copenhagen and Frederiksberg (KF 12-009/04).

An average intravenous bolus of 74.3 MBq (range 65.8–79.9 MBq) of ^123^ I-PE2I (MAP-Medical Technologies Oy, Tikkakoski, Finland) was given, immediately followed by a constant infusion (mean 96.5 MBq; range 88.6–100.1 MBq) of ^123^ I-PE2I for 3 hours. The B/I (bolus infusion) protocol was similar in both healthy subjects and patients, with a bolus worth 2.7 hours (range 2.6–2.8 hours) of infusion (the B/I ratio).[17,18] Six SPECT acquisitions of 10-minutes duration each were obtained between 120 and 180 minutes post injection, resulting in typically 3–3.5 million counts (range 2.4–4.2 million counts) in the main energy window for the summed acquisitions. The total amount of counts in the downscatter energy window was approximately 35%–40% of the total counts in the main window.

**Figure 3:**
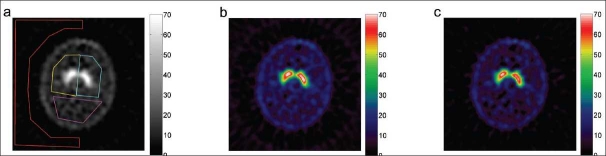
A reconstructed slice subject 3 (healthy) in Table 1. The regions of interest are shown in (a). The ROIs for the left stratium, right striatum, the reference region, and the region outside the brain are drawn in yellow, cyan, magenta, and red, respectively. The reconstructed slice is shown without downscatter correction (b) and with downscatter correction (c). Note the lower background and the improved contrast between the striatum and reference region. The amount of counts in the striata is slightly lower with correction, and the background outside the head is minimized by the correction.

**Figure 4:**
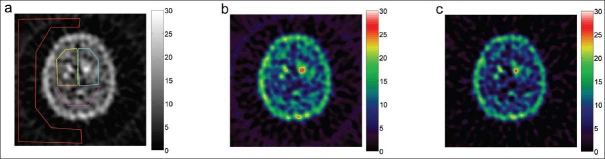
A slice of a reconstructed SPECT image for subject V (patient) in Table 1. The images shown correspond to those in Figure 3.

The performance of the downscatter correction was evaluated as image contrast after reconstruction. The noise properties of the reconstructed images were not investigated in detail in this paper. Image contrast was evaluated as (1) the striatal contrast and (2) the contrast between the intensities of the background outside the subject and the reference region (both of the latter regions are expected to have a uniform intensity). Specific binding ratio (SBR) is used as a measure for striatal contrast, which has the advantage that it is a clinically known and familiar quantity. The SBR is defined as the ratio of the specific striatal count concentration and the reference count concentration in the rest of the brain. Count concentrations are defined by c ≡ C/V and measured in counts/mL with C being the amount of counts and V the volume in milliliters. In order to minimize the subjectivity of drawing VOIs (volumes of interest), a method similar to the one devoveloped by Tossici-Bolt *et al*.[19] was used. This method is not sensitive to the partial volume effect, since all striatal counts are contained in a relatively large VOI covering a larger volume than the striatum. The VOIs were drawn on every slice for each individual subject where the striatum was visible, and the SBR for each striatum was calculated using the formula:

(4) $$\mathrm{SBR}\;\equiv \;\frac{c_{s}-\;c_{\mathrm{ref}}}{c_{\mathrm{ref}}}=\frac{1}{V_{s}}.\left(\frac{C_{\mathrm{VOI}}}{c_{\mathrm{ref}}}-V_{\mathrm{VOI}}\right)$$

where subscript S refers to the striatum, VOI to the volume of interest around the striatum, and the subscript ‘ref’ to the reference region. Identical VOIs were used for both reconstructions, with and without downscatter correction. In order to ensure that all the striatal counts were contained in the large VOI, an extra top and bottom slice with VOIs were added. Examples of the VOIs are shown in Figures 3a and 4a. A standard striatal volume of 11.2 mL was assumed.[19] However, due to individual variations, this might be significantly different from the volume measured by a structural MRI scan.[20] For this reason, some caution is needed before interpreting the SBR values determined in this way. In this study, however, the improvement in SBR by downscatter correction is important, and since the striatal volume is a constant scaling factor in Equation 4, it cancels out in the calculation of the relative improvement in SBR.

**Table 1:** Results of the evaluation for five healthy subjects (1–5) and five patients (I–V).

*Subject*	*k*	*SBR left*	*%*	*SBR right*	*%*	*Background/reference*
1.	1.159	6.17/7.35	19.2	6.14/7.07	15.1	0.162/0.008
2.	1.168	9.22/11.03	19.7	8.76/10.43	19.0	0.134/0.006
3.	1.170	10.14/12.61	24.4	9.72/12.28	26.4	0.152/–0.014
4.	1.163	8.45/10.70	26.6	9.13/11.32	24.1	0.152/–0.013
5.	1.155	9.25/11.46	23.9	7.91/9.93	25.6	0.133/–0.003
I.	1.179	2.11/2.28	7.9	2.06/2.31	12.3	0.119/–0.013
II.	1.174	3.99/4.66	16.9	5.50/6.61	20.2	0.117/0.004
III.	1.180	1.43/1.23	–13.5	1.72/2.02	17.9	0.141/0.007
IV.	1.181	5.30/5.69	7.3	4.38/4.50	2.7	0.155/0.004
V.	1.177	1.25/1.21	–3.1	1.57/1.42	–10.0	0.202/0.006

In the numerical fields where two values are separated by a slash, the first value is without downscatter correction (*k* = 0) and the second value is with downscatter correction (*k* = 1.1). The last column shows the contrast between background and the reference region. Uncertainty due to the amount of counts in SBR is of the order of 0.1. The percent symbol indicates the column with the relative improvement due to the downscatter correction

**Figure 5:**
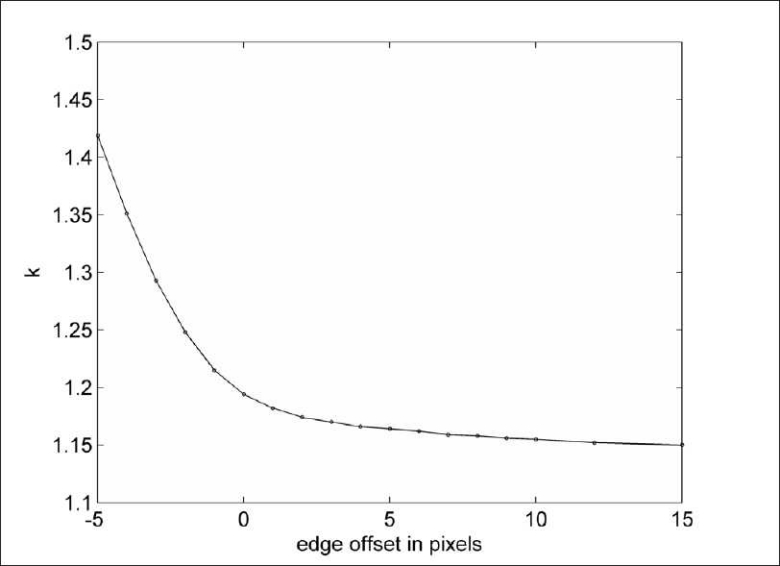
The calculated weighting factor k for subject 3 as a function of the edge-offset. Within the subject (offset<0) the assumption of no primary photon counts for the calculation of *k* is not valid.

For comparison with the I-123 source experiments, the weighting factor in each of the 10 subjects was determined by minimizing the background outside the subject. There should be no counts, neither primary photons nor scattered primary photons, *outside* the subject in the corrected projection data, i.e., J = 0 in Equation 1. By using the counts in the region outside the subject only the weighting factor can be determined by applying Equation 2. The background region for determining *k* was drawn well outside the head limits, as defined by expanding Chang’s attenuation map radially [Figure 2c] by three pixels (= 7 mm). The weighting factor *k* was calculated with Equation 2 applied to the raw projection data. For one subject, the edge threshold was varied in order to investigate its influence on *k* [Figure 5]. Differences between subject groups were *t*-tested, and the stated *P*-values were calculated by standard two-tailed *t*-tests. For the independent *t*-test, equal variance was not assumed.

## Results

**Figure 6:**
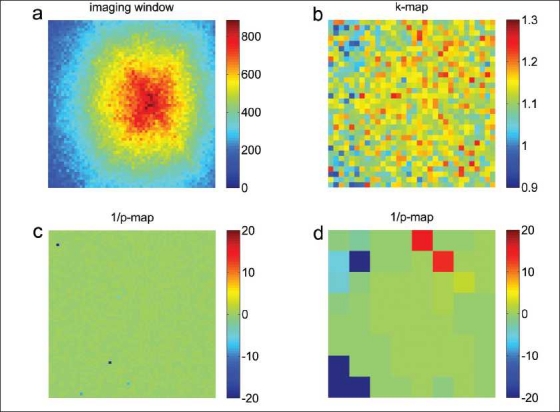
Projection data acquired by placing a 6-mm lead-shielded I-123 source in the SPECT scanner: (a) for energy window 1 (143–175 keV) in a 64 × 64 matrix size, data for energy window 2 (184–216 keV) looks similar to (a) (not shown); (b) the ratio (k map) of the two energy windows in a 32 × 32 matrix size; (c) 1/P maps in a 64 × 64 (c) and (d) 8 × 8 matrix size. The P-values correspond to Bonferroni-corrected double-sided significance levels. A 1/P value of 20 corresponds to a significance level of 5%. Negative P-values indicate negative differences.

Figure 6 shows the dataset for the lead-shielded I-123 and the 90° camera configuration (similar results were obtained for the two other configurations of 210° and 330°). The 1/*P* maps show almost no significant differences from the global value of *k*. To the left of the *k* map, relatively far away from the source, there may be a larger area with a somewhat lower value of *k*. In a 8 × 8 matrix this is also indicated by four pixels, with a significant negative difference. As for the I-123 source placed outside the scanner, the maps did not show any significant difference of *k* from the global value.

The weighting factor *k* was calculated with Equation 2 for each of the three camera configurations. For the shielded source in the scanner, the respective values of *k* were 1.123, 1.132, and 1.122. The total number of counts in the primary window was 1.7, 1.1, and 1.7 million counts, respectively. The uncertainty in *k* due to Poisson statistics was less than 0.002.

Similarly, for the source placed outside the scanner, the values of *k* were 1.107, 1.109, and 1.115 for the three angles. The total number of counts in the primary window was 0.37, 0.39, and 0.24 million counts. The uncertainty in *k* due to Poisson statistics was less than 0.003.

Based on these results, a *k*-value of 1.1 was chosen for the reconstruction of the subject data. The results for the subjects are listed in Table 1, and an example of a reconstructed slice of a healthy subject and a patient are shown in Figures 3 and 4, both with and without downscatter correction. Figures 3a and 4a show the manually drawn regions of interest for determining the specific binding ratio and the contrast between the background and reference region.

For comparison with the I-123 source experiments, the weighting factor *k* was also determined in the subjects by minimizing the background. It was found to be slightly higher than 1.1 and significantly different (*P*<.005) between healthy subjects and patients, with ranges of 1.155–1.170 and 1.174–1.181, respectively. The uncertainty in k, caused by the limited amount of counts, was 0.002 for all subjects.

The background in the image was effectively removed by the downscatter correction for all subjects [Figures 3c and 4c]. Downscatter (with *k*=1.1) and non-downscatter-corrected images are visually comparable, but show improved contrast. Because of the energy window subtraction, the mean amount of counts in the reference region, the background, and (in lesser degree) in the striatal region is decreased. This is because the reduction in mean counts in the striatal region is comparatively smaller than that in the reference region.

The contrast between background outside the subject and the reference region decreased from 0.15 ± 0.02 to 0.00 ± 0.01 (mean ± SD). A *t*-test revealed a significant difference (*P*<.005) between the SBR, both with and without downscatter correction for each striatum in healthy subjects. The uncertainty in SBR due to the limited amount of counts is approximately 0.1 or less. The difference in SBR was not significant (*P*>.2) for the patient group. SBR for the healthy subjects was increased by 23% ± 3% and 22% ± 5% (mean ± SD) for the left and right striatum, respectively. The relative SBR change in the striatum for patients ranged from –13.5% to 16.9% (left striatum) and from –10.0% to 20.2% (right striatum). Linear regression without intercept revealed a slope of (1.21 ± 0.01) between corrected and uncorrected SBRs for all subjects and for both left and right striatums in Table 1. The correlation coefficient was 99.8%.

**Figure 7:**
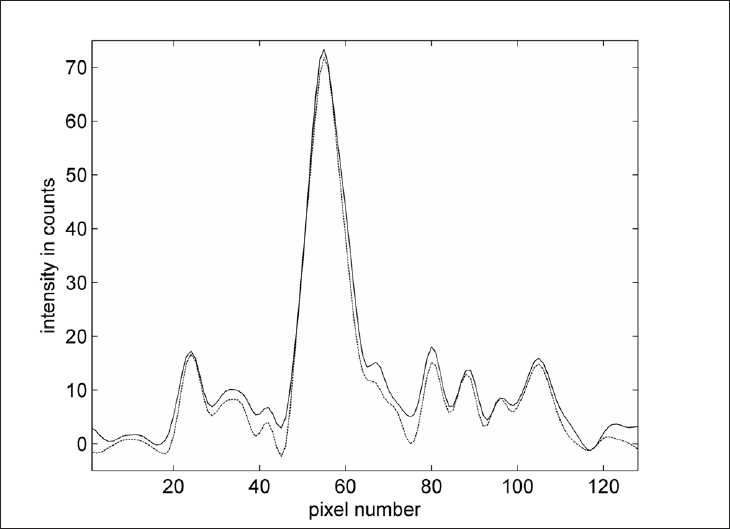
Intensity profile from front to the back of the head through the maximum of the left striatum of subject 3 [Figure 3]. The solid line is without downscatter correction, and the dashed line is with downscatter correction. Maximum intensities are similar, but the intensities are lower in the brain regions outside the striatum and close to zero outside the head.

Figure 7 shows an intensity profile though the left striatum of the healthy subject, both with and without downscatter correction. In a typical patient study, the amount of counts in the downscatter window is approximately 35%–40% of the amount of counts in the main window, which is comparable to the value reported by Du *et al*.[21]

## Discussion

A possible explanation for the small differences in *k* between the three camera configurations and the two setups with the iodine source could be the uncertainty in the calibration of the camera heads, the electronic noise, or a small geometrical dependency of *k*.

Inspection of the images in Figure 6 reveals a fairly uniform value of *k*. Smaller values of *k* are possibly observed in areas of no importance, far away from the source. Similar results are observed when the iodine source is placed outside the scanner (images not shown). This indicates that the assumption of spatial invariance is within reason. More counts, however, might reveal more significant differences.

Figure 5 shows the dependency of calculation of *k* on the edge offset for a healthy subject. The determined weighting factor increases with decreasing edge offset. The assumption of no primary photons is not valid close to the edge and this results in a too high a value of *k*. The value of *k* determined at a distance of 3 pixels (edge offset) to the head for a 2-pixel thick layer was calculated as 1.245 ± 0.009. This indicates that the *k* factor is slightly higher for projection lines going through the head. Source–detector distance dependency of the downscatter count rate has been reported before.[2,3] Should this distance dependency be energy dependent, this might explain the distance dependency of weighting factor *k*.

The small but statistically significant difference in *k* between healthy subjects and patients might be caused by more uniform distribution of activity in the brain in patients.

Downscattered photons are not collimated and therefore a sinogram of these photons (not shown) has very limited visible structure and subtraction of the downscatter energy window has to be performed *before* reconstruction. Downscatter correction is not necessary if medium energy collimators are used, since the amount of detected downscattered high-energy photons is negligible; however, this is at the cost of a loss in sensitivity.

The largely improved SBRs for healthy subjects make discrimination between healthy subjects and patients easier. The effect of the novel result of a higher value (compared to one) of the weighting factor on the SBR is not profound, though it is not completely negligible. In healthy subjects with an SBR of approximately 10, the SBR compared to the calculated SBR for *k*=1 is increased by 0.3 for *k*=1.1 and 0.6 for *k*=1.2.

The chosen downscatter window is on the high-energy side of the backscatter peak, while the main window is placed on the low-energy side of the backscatter peak. A downscatter window around a higher energy will result in a higher value for the weighting factor. With the energy windows used in this paper, the weighting factor is close to one because the maximum of the backscatter peak is situated between the two window positions.

Contrast might be improved even more by applying a scatter correction for the primary photons. This might be included by one or more energy windows below the main window, as in the dual-energy window scatter correction for Tc-99m[8] (later adapted for I-123 by Luo *et al*.[22]). An extra complication is the contribution of downscattered photons to the scatter energy window. The weight for the downscatter window needs to be reduced in this case.

Downscatter correction by energy window subtraction can easily be performed and is chosen because other techniques, such as the TDCS (transmission-dependent convolution subtraction) technique, demand advanced postprocessing.[9] Add to that the fact that the downscatter correction in the TDCS technique is often added as a rather crude constant scatter fraction.[10,11] The triple-energy window method[7] is straight forward, but suffers from noise sensitivity[23] for low count SPECT-studies. If a broad downscatter window is used, no noise issues are caused by the correction.

## Conclusion

Septal penetration of high-energy photons reduces the contrast of I-123 SPECT images if low-energy collimators are used, but it can be corrected in a simple and effective way by subtraction of a second (higher) energy window from the raw emission data. Two novel methods for determining the weight of the second energy window have been presented in this article, the first based on phantom work and the second on the minimization of the background in the projection images before reconstruction.

The value of the weighting factor was found to be slightly higher than one, a consequence of the structure of the downscatter energy spectrum above the main window. In clinical practice, a spatially invariant weighting factor with a value of *k*=1.1–1.2 (or experimentally determined on-site) can be used. Correcting for high-energy photons significantly improves the contrast between high- and low-count regions. In the case of SPECT brain studies of the dopamine transporter with the PE2I tracer, the contrast was improved by more than 20% in healthy subjects.
